# High-Oxygen-Barrier Multilayer Films Based on Polyhydroxyalkanoates and Cellulose Nanocrystals

**DOI:** 10.3390/nano11061443

**Published:** 2021-05-30

**Authors:** Beatriz Melendez-Rodriguez, Sergio Torres-Giner, Inmaculada Angulo, Maria Pardo-Figuerez, Loïc Hilliou, Jose Manuel Escuin, Luis Cabedo, Yuval Nevo, Cristina Prieto, Jose Maria Lagaron

**Affiliations:** 1 Novel Materials and Nanotechnology Group, Institute of Agrochemistry and Food Technology (IATA), Spanish Council for Scientific Research (CSIC), 46980 Valencia, Spain; beatriz.melendez@iata.csic.es (B.M.-R.); storresginer@upv.es (S.T.-G.); mpardo@iata.csic.es (M.P.-F.); cprieto@iata.csic.es (C.P.); 2 Gaiker Technology Centre, Basque Research and Technology Alliance (BRTA). Parque Tecnológico de Bizkaia, edificio 202, 48170 Zamudio, Bizkaia, Spain; angulo@gaiker.es; 3 Bioinicia R&D, Bioinicia S.L., 46980 Valencia, Spain; 4 IPC/I3N, Institute for Polymers and Composites, Department of Polymer Engineering, University of Minho, 4800-058 Braga, Portugal; loic@dep.uminho.pt; 5 Tecnopackaging S.L., Poligono Industrial Empresarium, 50720 Zaragoza, Spain; info@tecnopackaging.com; 6 Polymers and Advanced Materials Group (PIMA), School of Technology and Experimental Sciences, Universitat Jaume I (UJI), 12071 Castellón, Spain; lcabedo@uji.es; 7 Melodea Bio-Based Solutions, Faculty of Agriculture-Hebrew University, Rehovot 76100, Israel; yuval@melodea.eu

**Keywords:** PHBV, nanocellulose, multilayers, barrier films, packaging

## Abstract

This study reports on the development and characterization of organic recyclable high-oxygen-barrier multilayer films based on different commercial polyhydroxyalkanoate (PHA) materials, including a blend with commercial poly(butylene adipate-*co*-terephthalate) (PBAT), which contained an inner layer of cellulose nanocrystals (CNCs) and an electrospun hot-tack adhesive layer of poly(3-hydroxybutyrate-*co*-3-hydroxyvalerate) (PHBV) derived from cheese whey (CW). As a result, the full multilayer structures were made from bio-based and/or compostable materials. A characterization of the produced films was carried out in terms of morphological, optical, mechanical, and barrier properties with respect to water vapor, limonene, and oxygen. Results indicate that the multilayer films exhibited a good interlayer adhesion and contact transparency. The stiffness of the multilayers was generally improved upon incorporation of the CNC interlayer, whereas the enhanced elasticity of the blend was reduced to some extent in the multilayer with CNCs, but this was still much higher than for the neat PHAs. In terms of barrier properties, it was found that 1 µm of the CNC interlayer was able to reduce the oxygen permeance between 71% and 86%, while retaining the moisture and aroma barrier of the control materials.

## 1. Introduction

Packaging materials based on biopolymers that can biodegrade in both industrial and home compost conditions currently represent an alternative to solve the environmental issue of plastic accumulation [1,2]. Polyhydroxyalkanoates (PHAs) are thermoplastic biopolyesters produced by various microorganisms, mainly bacteria, during fermentation of sugar or lipids under famine conditions as energy-reserve inclusions in the cytoplasm [3]. The most widely studied PHA is poly(3-hydroxybutyrate) (PHB), whose thermal and mechanical characteristics are similar to polypropylene (PP) [4]. However, PHB is brittle and presents a poor processing window due to its high crystallinity and low thermal stability. For these reasons, PHB is being progressively replaced by its poly(3-hydroxybutyrate-*co*-3-hydroxyvalerate) (PHBV) and poly(3-hydroxybutyrate-*co*-4-hydroxybutyrate) P(3HB-*co*-4HB) copolymers [5,6]. PHAs are not only bio-based but also industrially compostable and biodegradable in the environment [7,8]. Thus, PHAs show a great potential to replace conventional polyolefins for packaging applications [9].

Furthermore, PHA microbial polyesters show relatively high water vapor and moderate oxygen barrier properties [10]. In this scenario, nanocellulose can play an important role in packaging applications as a novel and sustainable oxygen barrier material [11,12,13]. On the one hand, cellulose is a naturally occurring polymer that is stored in plant cell walls, which can be isolated from various wooden and nonwooden sources by different chemical and mechanical treatments [14]. On the other hand, it is fully biodegradable in the environment [15,16]. For example, Qi et al. [17] reported complete biodegradation of cellulose films in soil at about 30 °C within 1 month. Within the different kinds of celluloses, there are three main categories, namely, cellulose nanofibrils (CNFs), which contain amorphous and crystalline regions [18], cellulose nanocrystals (CNCs), and bacterial nanocellulose (BNC), with the latter still under development for large-scale industrial production [19,20]. In the case of CNCs, these can be obtained by processing cellulose under carefully controlled acidic treatment conditions [21]. These cellulosic particles show diameters in the 5–20 nm range, whereas lengths range from 100 to 400 nm. Furthermore, CNCs have high self-assembly properties, which allows the production of continuous materials such as high-quality self-supporting transparent films and coatings that are habitually termed “nanopapers” [22]. These nanocellulose films exhibit very low gas permeability, which makes them perfect candidates for compostable high-gas-barrier packaging applications [23]. However, nanopapers are also highly hydrophilic due to the presence of a large number of hydroxyl groups (–OH), which represents a disadvantage for their use in packaging in moist environments. 

So far, some research studies have dealt with the improvement of the barrier properties of nanopapers in high-humidity conditions, for instance, by dispersing CNCs in hydrophobic polymer matrices [24], by performing surface modifications on CNC films [25], and by crosslinking treatments [26]. Another innovative strategy is the development of multilayer systems that can protect the CNC layers with hydrophobic polymers as outer layers, such as PP and polyethylene terephthalate (PET) [27,28]. Multilayers are structures widely used in the food packaging industry that are created by assembling a different number of layers, typically between three and nine, where each layer provides a particular performance to the whole system. Typically, the outer layers, also called structural layers, provide food contact, printability, and mechanical and moisture resistance, whereas the intermediate layers provide the necessary barrier to gases and organic vapors to preserve food quality and safety [29]. Therefore, the high performance of multilayer films makes them very suitable for use in the packaging industry, especially for extending goods shelf life [30].

Today, there are different methods for the preparation of multilayers, for instance, layer-by-layer (LbL) assembly [31], co-extrusion [32], co-injection stretch blow molding [33], lamination [34], metallization [35], and coatings by plasma [36] or solvent casting [37]. More recently, electrospinning has emerged as an innovative technique to generate polymer mats composed of fibers with diameters ranging from micro- to nanoscale via the action of high-electric fields, allowing the formation of coatings and monolayers of interest in the packaging industry [38,39,40]. In this context, Figueroa-Lopez et al. [41] developed electrospun PHBV fibers containing eugenol as potential antimicrobial monolayers in multilayer structures for food packaging. Similarly, Cherpinski et al. [42] developed electrospun coatings of biopolyesters to improve the barrier properties and water resistance of paper packaging. Furthermore, electrospinning allows the incorporation of functional ingredients into the polymer fibers since it is carried out at room temperature, opening up novel opportunities in active and bioactive packaging [43]. Additionally, the application on the electrospun fiber mats of a thermal post-treatment below the biopolymer’s melting temperature (T_m_), also called annealing, yields the formation of continuous films, so-called biopapers [44,45,46]. Electrospinning can favor the preservation of thermolabile and volatile substances in the film layers since the annealing process of the electrospun fibers is carried out at lower temperatures and shorter times in comparison with the melting routes [44,47]. Moreover, these annealed electrospun mats, so-called biopapers, can also originally perform as hot-tack (HT) interlayers via a mechanism of interfiber coalescence, adhering different film layers without the need for tie layers [48].

Previous studies in our laboratory developed multilayers for their use in both rigid and flexible packaging with antimicrobial and vapor barrier properties, in which electrospun PHA fibers with active substances and CNC coatings were used as intermediate layers to provide these properties [41,49]. Following this concept, the present study focuses on the development of new multilayer designs for use as a high-oxygen-barrier film. To this end, a film prepared with three different types of PHA-based substrates, i.e., a commercial PHBV film, an extruded PHBV film, and a PHA-based blend film with poly(butylene adipate-*co*-terephthalate) (PBAT), was coated with a CNC layer. In parallel, another film was coated with electrospun industrial biowaste-derived PHA fibers, used as HT. Thereafter, the two coated layers were assembled together by lamination, and their morphological, optical, mechanical, and barrier properties were evaluated to assess the potential application of the resulting multilayer films in organic recyclable food packaging.

## 2. Materials and Methods

### 2.1. Materials

The CNC commercial aqueous solution was supplied by Melodea Ltd. (Rehovot, Israel) with a concentration of 2 wt.%, yielding a pH of 4.5. The CNC suspension was stored at 4 °C as received to be used within a 1-month period. 1-Butanol, reagent grade with 99.5% purity, and d-limonene, with 98% purity, were both obtained from Sigma Aldrich S.A. (Madrid, Spain). Chloroform of 99.8% purity was purchased from Panreac S.A. (Barcelona, Spain). The food contact primer, Loctite Liofol PR1550, was supplied by Henkel Ibérica S.A. (Barcelona, Spain).

A PHBV copolyester was obtained from fermented cheese whey (CW), a waste of the dairy industry. Further details about the material and its synthesis can be found elsewhere [50]. The PHBV copolymer was purified using the chloroform-based extraction method reported previously [45,46,47]. The 3HV content of the copolymer was 20 mol.% as determined by gas chromatography (GC) using the method described by Lanham et al. [51] in a Bruker 430-GC gas chromatograph equipped with a flame ionization detector (FID) and a BR-SWax column (60 m, 0.53 mm internal diameter, 1 mm film thickness, Bruker, Torrance, CA, USA).

A commercial film of PHBV containing 8 mol.% 3HV, so-called PHBV8, with a thickness of 25 µm, was purchased from Goodfellow Cambridge Limited (Huntindgon, UK) as grade BV301025. 

The PHA blend compound containing 50 wt.% PHB and 50 wt.% PBAT was produced by Tecnopackaging (Zaragoza, Spain). For this, the PHB grade (Biomer^®^ P309) was supplied by Biomer (Krailling, Germany). This grade has a melt flow index (MFI) of 10 g/10 min at 180 °C for a load of 2.16 kg. A film blowing grade of PBAT (Ecoflex^®^ F blend C1200) was supplied by BASF (Ludwigshafen am Rhein, Germany). This grade has an MFI of 2.5–4 g/10 min at 190 °C for a load of 2.16 kg and was claimed to be fully compostable by the manufacturer. Film blowing of the blend was also performed by Tecnopackaging using film blowing extrusion equipment (LABTECH LF400 from Techlab Systems S.L., Lezo, Spain). The parameters used in the machine were as follows: max. bubble diameter of 350 mm, variable blowing speed, twin-screw extruder LE25-30/C, large 2.4 m high film tower, pneumatically operated film nip rolls, screw speed infinite variable from 0 to 300 rpm, and motorized adjustment of film tower height. The set parameters of the film blowing experiments were as follows: screw speed of 65 rpm, screw pressure of 196 bar, screw temperature profile of 170 °C/170 °C/168 °C/168 °C, superior roll speed of 1.8 m/min, collection roll speed of 2.7 m/min, and tower height of 1500 mm. The resulting blown film, so-called PHB Blend, had a thickness of around 60 µm and a film width of 250 mm.

A noncommercial PHBV film, so-called PHBV2, was obtained by delamination of a two-layer co-extruded film blowing film. The procedure reported earlier by Cunha et al. [52] was followed to obtain the bilayer film made of PHBV_int_/PBAT_out_ (PBAT as external layer and PHBV as internal layer). The PHBV used was ENMAT Y1000P, produced by Tianan Biologic Materials (Ningbo, China), whereas the PBAT grade used was the same grade as described above. In the case of PHBV, the 3HV fraction was 2 mol.% with a density of 1.25 g/cm^3^ and a molecular weight (M_W_) of 2.8 × 10^5^ g/mol. The bilayer coextruded film was performed at the University of Minho (Portugal) in a laboratory blown film extrusion line (Periplast, Portugal), which was configured with an extrusion/co-extrusion head and die for the production of bilayered films from combined grades. The set temperature profile in both extruders was 150 °C/155 °C/155 °C from hopper to screw tip, and the head and die were kept at 155 °C/155 °C/160 °C. The screw speed was set at 46 rpm, which corresponded to outputs of approximately 3 kg/h. All materials were dried for 24 h at 60 °C before processing. During co-extrusion, both fan speed and air ring aperture were kept constant (to maintain similar cooling conditions), while both the blow-up ratio (BUR) and the take-up ratio (TUR) were varied to produce films with thicknesses ranging roughly from 70 to 150 mm. Since the bilayer film showed easy delamination, the PHBV layer was separated from the PBAT layer, and the former was used as substrate in this study. The PHBV2 film had a thickness of ca. 70 µm.

### 2.2. Application of the CNCs

A layer of CNCs was applied using lab or pilot applicators (see below) on one side of the three film substrates, that is, PHBV8, PHBV2, and PHB Blend. Before applying CNCs, a corona treatment (100 watt∙cm^2^/min) was applied on the three substrates in order to make them more hydrophilic, decreasing the contact angles of the surfaces. A food contact primer layer containing a wetting agent to facilitate homogeneous coating was applied on the substrates prior to coating with CNCs since it was seen to improve the adhesion between the substrates and the CNC layer. The food contact primer and wetting agent were mixed in an IKA Eurostar 6000 mixer (IKA^®^-Werke GmbH & Co. KG, Staufen, Germany) at low speed (200 rpm) to avoid bubbles. During processing, it was observed that the primer allowed an increase in the stability of the layers at elevated temperatures. In addition, the CNC layer was easier to coat using the primer.

The multilayers of PHBV8 and PHBV2 were laminated at a lab scale, whereas the PHB Blend was laminated at a pilot scale. At a lab scale, the food contact primer was applied on the surface of the treated films by means of an automatic film applicator (Zehntner ACC378.100) with a profile rod coater of 6 μm wet thickness and dried in an oven at 90 °C for 1 min. On top of the primer, the CNC solution was coated using the same automatic film applicator (Zehntner ACC378.100) with a profile rod of 100 μm wet thickness and a speed of 5 mm/sec. The drying temperature in the oven was 90 °C for 15 min, and the final thickness of the CNC layer was ca. 1 μm. At a pilot scale, the primer was applied onto the PHB Blend by a meter bar head with a profile rod of 6 μm in a Rotary Koater (ROKO) Model 30-30-01 equipment (TMI Machine Testing Inc., New Castle, DE, USA) and dried at 50 °C. The CNC solution was applied on top of the primer with a profile rod of 50 μm. Drying was performed in continuous in the lamination equipment at 90 °C at 1 m/min to ensure complete drying of the CNC solution.

### 2.3. Electrospinning of Food Waste Derived PHBV

In order to improve the adhesion between the uncoated and the CNC-coated substrates, an electrospun fiber mat of the PHBV derived from industrial cheese whey was applied on the uncoated substrate. The electrospun fibers were used as HT due to the fact that the high roughness of the ultrathin fiber mat was found to facilitate film bonding upon annealing.

Prior to electrospinning, the purified PHBV powder was dissolved at 8 wt.% in a mixture of chloroform and butanol 75:25 (*w/w*) under magnetic stirring for 24 h at 50 °C. This solvent mixture is, to the best of our knowledge, the most optimal and sustainable organic solvent system able to successfully electrospin PHAs. The PHBV solution was processed by electrospinning using a high-throughput dual polarization Fluidnatek^®^ LE-500 pilot-plant device containing a roll-to-roll system manufactured by Bioinicia S.L. (Valencia, Spain). The equipment was operated with a motorized multi-needle injector, scanning vertically toward the different substrate collectors (PHBV8, PHBV2, and PHB Blend) at 25 °C and 40% RH. The conditions were optimized for the solution, being set at a flow rate of 45 mL/h, 22 kV of voltage, and 30 cm of needle-to-collector distance.

### 2.4. Lamination

**Figure 1:**
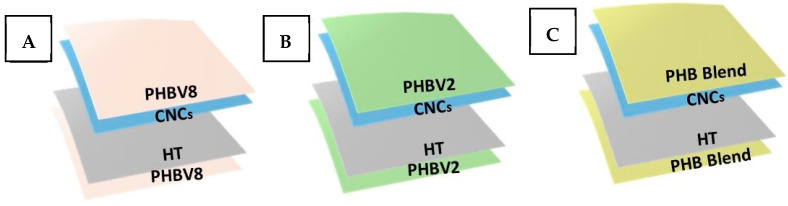
Schematics of the multilayer films of (**A**) poly(3-hydroxybutyrate-*co*-3-hydroxyvalerate) containing 8 mol.% 3-hydroxyvalerate (PHBV8), (**B**) poly(3-hydroxybutyrate-*co*-3-hydroxyvalerate) with 2 mol.% 3-hydroxyvalerate (PHBV2), and (**C**) poly(3-hydroxybutyrate) with poly(butylene adipate-*co*-terephthalate) blend (PHB Blend). The electrospun biowaste-derived poly(3-hydroxybutyrate-*co*-3-hydroxyvalerate) with 20 mol.% 3-hydroxyvalerate mat was used as a hot-tack (HT) coating on one layer, while the cellulose nanocrystal (CNC) layer was added on the other one.

Lamination of the coated layers was carried out using a Reliant Powerbond equipment (Reliant Machinery Ltd., Luton, UK), depositing the samples over the equipment conveyor belt that traveled through the oven at a speed of 5 m/min at 140 °C for 20 s. The resultant multilayer samples based on PHBV2 and PHB Blend had an average thickness in the 130–150 μm range, whereas that based on the PHBV8 presented a thickness of 50 μm. The final structures are depicted in Figure 1. Equivalent multilayers without CNCs were obtained, in the same conditions, as controls.

### 2.5. Characterization

#### 2.5.1. Scanning Electron Microscopy

The cross-section of the three multilayers was observed by scanning electron microscopy (SEM) using an S-4800 device from Hitachi (Tokyo, Japan). The multilayers were cryo-fractured by immersion in liquid nitrogen and, then, fixed to beveled holders using conductive double-sided adhesive tape. The samples were sputtered with a mixture of gold/palladium under vacuum prior to observation. An accelerating voltage of 10 kV was used, and the estimation of the dimensions was performed by means of the Aperture software from Apple (Cupertino, CA, USA) using a minimum of 20 SEM micrographs in their original magnification.

#### 2.5.2. Transparency

The light transmission of the multilayers was determined in specimens of 50 mm × 30 mm by quantifying the absorption of light at wavelengths between 200 and 700 nm in an ultraviolet–visible (UV–Vis) spectrophotometer VIS3000 from Dinko Instruments (Barcelona, Spain). The transparency (*T*) and opacity (*O*) were calculated using Equations (1) [53] and (2) [54], respectively.

(1) $$T=\frac{A_{600}\;}{L},$$

(2) $$O=A_{500\;}\times L,$$

where A_500_ and A_600_ are the absorbance values at 500 and 600 nm, respectively, and L is the film thickness (mm).

#### 2.5.3. Color Measurements

The multilayer color was determined using a chroma meter CR-400 (Konica Minolta, Tokyo, Japan). The color difference (∆E*) was calculated, as defined by the Commission Internationale de l’Eclairage (CIE), using Equation (3) [55].

(3) $$∆E^{*\;}={\left[{\left(∆L^{*}\right)}^{2\;}+{\left(∆a^{*}\right)}^{2}+{\left(∆b^{*}\right)}^{2}\right]}^{0.5},$$

where ∆L*, ∆a*, and ∆b* correspond to the differences in terms of lightness from black to white, color from green to red, and color from blue to yellow, respectively, between the test multilayers with CNCs and the control samples (without CNCs). Color change was evaluated using the following assessment: unnoticeable (ΔE*  < 1), only an experienced observer can notice the difference (ΔE*  ≥ 1 and  < 2), an unexperienced observer can notice the difference (ΔE*  ≥ 2 and  < 3.5), clear noticeable difference (ΔE*  ≥ 3.5 and  < 5), and the observer can notice different colors (ΔE* ≥ 5) [56].

#### 2.5.4. Mechanical Tests

Tensile tests of the multilayers were conducted in a universal testing machine Shimatzu AGS-X 500N (Shimatzu, Kyoto, Japan) at room temperature with a crosshead speed of 10 mm/min. Dumbbell samples according to ASTM D638 (Type IV) standard were die-cut from the multilayer assembly both in the machine direction (MD) and in the transversal direction (TD). Samples were conditioned to the test conditions at 40% RH and 25 °C for 24 h prior to tensile assay. At least six samples were tested for each material, and the average values of the mechanical parameters and standard deviations were reported.

#### 2.5.5. Permeance Tests

The water vapor permeance (WVP) of the multilayers was determined using the gravimetric method ASTM E96-95 in triplicate. The control samples were cups with aluminum films to estimate solvent loss through the sealing. For this, 5 mL of distilled water was placed inside a Payne permeability cup (diameter of 3.5 cm) from Elcometer Sprl (Hermallesous-Argenteau, Belgium). The multilayers were not in direct contact with water but exposed to 100% RH on one side and secured with silicon rings. They were placed within a desiccator, sealed with dried silica gel, at 0% RH in a cabinet at 25 °C. WVP was calculated from the regression analysis of weight loss data vs. time, and the weight loss was calculated as the total loss minus the loss through the sealing. 

For limonene permeance (LP), the procedure was similar to that described above for WVP with the difference that 5 mL of d-limonene was placed inside the Payne permeability cups, which were placed under controlled room conditions of 25 °C and 40% RH. 

The oxygen permeance (OP) coefficient was derived from the oxygen transmission rate (OTR) measurements that were recorded using an Oxygen Permeation Analyzer M8001 from Systech Illinois (Thame, UK) at 20% RH and 25 °C, in duplicate. The samples were purged with nitrogen, before exposure to an oxygen flow of 10 mL/min. The exposure area during the test was 5 cm^2^ for each sample. 

### 2.6. Statistical Analysis

The optical, mechanical, and barrier properties were evaluated through analysis of variance (ANOVA) using STATGRAPHICS Centurion XVI v 16.1.03 from StatPoint Technologies, Inc. (Warrenton, VA, USA). Fisher’s least significant difference (LSD) was used at the 95% confidence level (*p* <0.05).

## 3. Results and Discussion

### 3.1. Morphology of the Multilayers

**Figure 2:**
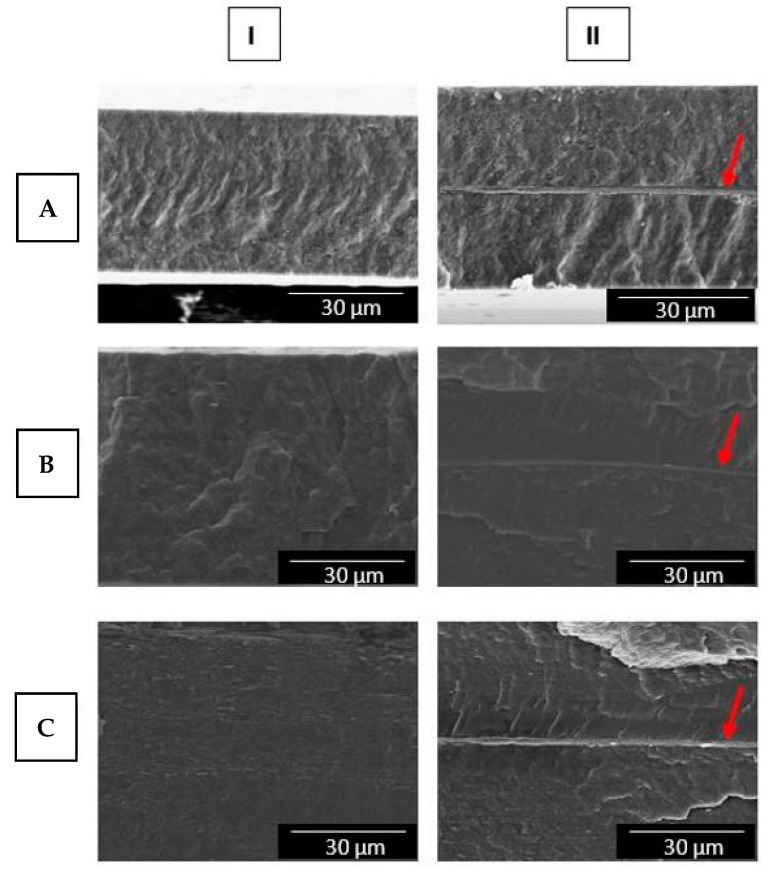
Scanning electron microscopy (SEM) micrographs of the cross-sections of the multilayer films of: (**A**) poly(3-hydroxybutyrate-*co*-3-hydroxyvalerate) containing 8 mol.% 3-hydroxyvalerate (PHBV8), (**B**) poly(3-hydroxybutyrate-*co*-3-hydroxyvalerate) with 2 mol.% 3-hydroxyvalerate (PHBV2), and (**C**) poly(3-hydroxybutyrate) with poly(butylene adipate-*co*-terephthalate) blend (PHB Blend), without (**I**) and with cellulose nanocrystals (CNCs) (**II**) coating. Images were taken at 1500× with scale markers of 30 μm. Red arrows indicate the CNC layer.

The morphologies of the cross-sections of the three multilayers with and without CNCs analyzed by SEM are shown in Figure 2. The multilayers without CNCs, Figure 2IA–C, showed homogeneous surfaces without separation between the two layers. The HT could not be discerned in the SEM images, indicating good adhesion with the PHBV8, PHBV2, and PHB Blend layers, since all were based on PHA. When the CNCs were incorporated, this material was seen to form a continuous layer approximately of ca. 1 µm after cryofracture (see Figure 2IIA–C), thus supporting a good adhesion between the different layers.

The display of the CNC layer when coated in a multilayer system was previously reported [57,58]. The natural adhesive properties of electrospun polymers have also been reported when used as an interlayer in a multilayer system. Thus, previous works have shown the way in which annealed polymer fibers keep layers of different polymers together without the need for synthetic adhesives, maintaining the biodegradability and/or renewability of the final structure [48]. In addition to this advantage, improvements in optical and barrier properties have been reported when intermediate layers of electrospun fibers are used, as this technique allows the thickness to be controlled as required [59,60].

### 3.2. Optical Properties

**Figure 3:**
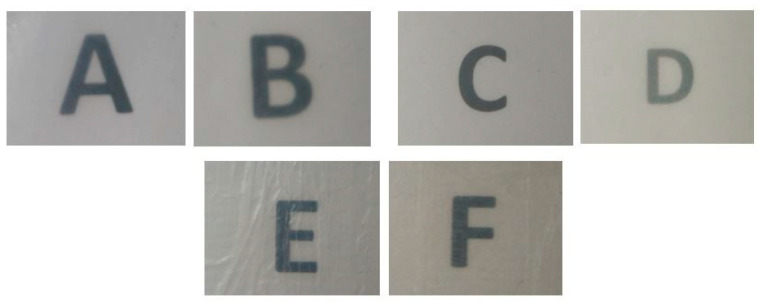
Contact transparency pictures of the multilayers made of (**A**) poly(3-hydroxybutyrate-*co*-3-hydroxyvalerate) containing 8 mol.% 3-hydroxyvalerate (PHBV8) without cellulose nanocrystals (CNCs), (**B**) PHBV8 with CNCs, (**C**) PHBV with 2 mol.% 3-hydroxyvalerate (PHBV2) without CNCs, (**D**) PHBV2 with CNCs, (**E**) poly(3-hydroxybutyrate) with poly(butylene adipate-*co*-terephthalate) blend (PHB Blend) without CNCs, and (**F**) PHB Blend with CNCs. Letters were placed underneath the films to assess their contact transparency.

The pictures of the resulting multilayers prepared with the three different substrates are displayed in Figure 3. All three substrates, regardless of whether the CNCs were present or not, showed a good contact transparency. More specifically, a slight yellowish tone was presented in the case of PHBV2 and PHB Blend substrates, whilst no color was observed in the PHBV8. These colors are intrinsic to the materials used, and the incorporation of CNCs and HT did not affect the final color of the multilayers, as both are colorless and the thickness used was likely not sufficient to have any influence on this.

The color parameters (a*, b*, L*) and the transparency (T) and opacity (O) values are reported in Table 1. All substrates presented positive values in a*, indicating that the multilayers tended to be red instead of green, while b* varied from negative values toward a blue tonality. With respect to L*, all the multilayers showed values around 90. The color differences between the multilayers when CNCs were added are also reported. While PHBV8 and PHB Blend presented differences that only an experienced observer would notice, PHBV2 showed clear noticeable differences, which could be ascribed to the fact that the film was more heterogeneous and, therefore, depending on the area chosen, its thickness could vary. Regarding the T value, the most transparent multilayers were PHB Blend, followed by PHBV8 and, finally, PHBV2. It should be noted that the addition of the CNC coating made all multilayers more transparent, meaning that the CNC coating resulted in a smoother surface of the substrate and, consequently, greater transparency. This homogeneity caused UV–Vis light to pass through the films without inducing light scattering. Lastly, all the multilayers showed similar low values of O, ranging between 0.02 and 0.2. Transparency is a key property in many types of food packaging, as visual inspection through the material is preferred by the consumers [61]. In this case, it can be stated that all tested multilayers provided a good level of transparency, which could be of interest when transparent packaging is required.

### 3.3. Mechanical Properties

The mechanical properties in both transversal direction (TD) and machine direction (MD) in terms of elastic modulus (E), tensile strength (σ_y_), elongation at break (ε_b_), and tensile toughness (T) of the multilayers were assessed, and the results are gathered in Table 2. The compositions with pure PHBV presented a stiff and brittle mechanical behavior, with elastic moduli above 2 GPa, elongation at break below 3%, and very low tensile toughness for both materials, being more remarkable for the sample with lower HV content when compared to the PHBV8. This mechanical behavior is in good agreement with the brittle nature of PHB and PHBV with low HV content [62]. Blending the PHB with PBAT resulted in a toughening effect, as derived from the considerable increase in the elongation at break and tensile toughness compared to the PHBV materials. However, this toughening effect entailed a decrease in the elastic modulus.

Generally speaking, it can be seen that the CNC coating did not significantly change the mechanical properties of the multilayers. However, a slight reinforcing effect could be inferred for some cases. For instance, the elastic moduli of PHBV2 in MD and PHB blend in both directions increased approximately 15% with the addition of the CNCs. On the other hand, the sample PHBV8 presented a decrease when incorporating the CNC layer. This was probably due to a delamination of the multilayer assembly upon tensile testing. This mechanical behavior is consistent with the literature and previous works of the group [63]. Thus, Cherpinski et al. [64] reported values of 2056.7 MPa, 21.0 MPa, and 5.9%, for E, σ_y_, and ε_b_, respectively, for a multilayer of CNFs with double-sided PHBV coatings prepared by electrospinning, which were quite similar to the neat PHBV. Moreover, previous work in our lab also showed no difference in mechanical properties between multilayers with a CNC coating and those without the CNC layer [49].

### 3.4. Barrier Properties

The permeance to water vapor (WVP), limonene (LP), and oxygen (OP) was measured in order to assess which multilayer system presented better barrier properties and how the addition of a CNC coating could affect their barrier performance. Table 3 shows the permeance values in terms of WVP, LP, and OP of the multilayers with and without CNCs. It can be seen that the PHB Blend showed the best barrier performance, showing values of 0.85 × 10^−11^ kg·m^−2^·Pa^−1^·s^−1^, 1.10 × 10^−11^ kg·m^−2^·Pa^−1^·s^−1^, and 3.90 × 10^−16^ m^3^·m^−2^·s^−1^·Pa^−1^, for WVP, LP, and OP, respectively. PHBV2 also showed good barrier properties, i.e., 0.90 × 10^−11^ kg·m^−2^·Pa^−1^·s^−1^, 2.02 × 10^−11^ kg·m^−2^·Pa^−1^·s^−1^, and 6.37 × 10^−16^ m^3^·m^−2^·s^−1^·Pa^−1^, for WVP, LP, and OP, respectively. Lastly, PHBV8 presented the lowest barrier properties, with values of 11.47 × 10^−11^ kg·m^−2^·Pa^−1^·s^−1^, 13.91 × 10^−11^ kg·m^−2^·Pa^−1^·s^−1^, and 57.81 × 10^−16^ m^3^·m^−2^·s^−1^·Pa^−1^, for WVP, LP, and OP, respectively. The PHBV8 presented the lowest barrier performance due to its lowest thickness and because it contained a higher fraction of 3HV in the copolymer composition [65,66]. In the case of the PHB Blend, the highest barrier values achieved were likely the result of a higher thickness, the presence of the PHB homopolymer [67,68], and perhaps a more favorable morphology of the film since this material was more flexible due to the elastomeric PBAT component. 

The excellent oxygen barrier properties imparted by CNCs are well known. However, at the same time, it is known that, when CNC is exposed to high-humidity conditions, these excellent properties decrease dramatically due to its hydrophilic nature. For this reason, the application of a CNC interlayer between moisture barrier polymers is considered as the most adequate method to overcome the biopolymer moisture sensitivity [64,69,70]. Furthermore, CNCs also provide flexibility and sealability to the final structure [71,72]. When CNCs were added to the multilayers, a small or null barrier improvement was seen for water and limonene, yet an improvement was clearly observed for oxygen. The permeance to oxygen gas was seen to decrease between approximately 71% and 86% for the different materials, with PHBV2 being the material with the highest barrier improvement. Therefore, this study further confirms that a barrier improvement to oxygen is provided by CNCs, which is in agreement with the previous literature. Thus, Le Gars et al. [73] reported a decrease in OP of about 90% in multilayers of polylactide (PLA) and CNCs (PLA/CNCs/PLA) compared to neat PLA. Similarly, Fotie et al. [74] prepared multilayers of five different polymers, i.e., PET, PLA, oriented polypropylene (OPP), PP, and PE with a 1 µm CNC interlayer, and, in all cases, an OP reduction between 90% and 100% was achieved after lamination. 

## 4. Conclusions

In this study, three different multilayer systems based on different commercial PHAs were assembled with a CNC interlayer and an HT layer made of PHBV fibers produced by electrospinning. The resultant structures were characterized in terms of morphological, optical, mechanical, and barrier properties. The SEM images showed good adhesion between the different layers, with no separation between them, along with a ca. 1 µm thick CNC coating. All the samples showed good contact transparency, and, while the substrates of PHBV2 and PHB Blend were slightly yellowish, the PHBV8 showed no color. In terms of mechanical properties, the PHB Blend exhibited, as expected, improved toughness and ductility compared to the other two multilayers, and, while these properties were reduced when CNCs were present in the structure, they were still much higher than for the pure commercial PHAs. All the multilayers showed improved barrier properties toward oxygen, while the water and limonene permeance remained largely unaffected. 

The multilayer systems presented in this study, especially the so-called PHB Blend, exhibited the best balance in all the measured properties. Despite the fact that the film components are known to lead a compostable packaging, a complete biodegradation study is currently underway in the films and will be reported elsewhere. Furthermore, the use of bio-based compostable materials to generate such structures, especially in the case of the hot-tack derived from food by-products, has been shown to not only offer significant advantages in the design of oxygen-sensitive food packaging technologies, but also contribute to the compliance with current trends toward a Circular Bioeconomy future for the overall food chain. 

## Figures and Tables

**Table 1:** Optical properties of the multilayer films of poly(3-hydroxybutyrate-*co*-3-hydroxyvalerate) (PHBV) containing 8 mol.% (PHBV8) and 2 mol.% 3-hydroxyvalerate (PHBV2) and poly(3-hydroxybutyrate) with poly(butylene adipate-*co*-terephthalate) blend (PHB Blend), with and without cellulose nanocrystals (CNCs).

Sample	a*	b*	L*	ΔE*	T	O
PHBV8	2.05 ± 0.05 ^a^	−3.07 ± 0.08 ^a^	90.98 ± 0.02 ^a^	-	11.51 ± 0.04 ^a^	0.03 ± 0.01 ^a^
PHBV8 with CNCs	2.41 ± 0.07 ^a^	−3.89 ± 0.05 ^b^	90.53 ± 0.02 ^a^	1.00 ± 0.02 ^a^	9.29 ± 0.03 ^b^	0.02 ± 0.01 ^a^
PHBV2	1.46 ± 0.07 ^b^	0.28 ± 0.02 ^c^	90.58 ± 0.03 ^a^	-	14.39 ± 0.07 ^c^	0.07 ± 0.02 ^a,b^
PHBV2 with CNCs	0.53 ± 0.02 ^c^	4.17 ± 0.07 ^d^	90.07 ± 0.05 ^a^	4.03 ± 0.03 ^b^	10.86 ± 0.05 ^d^	0.18 ± 0.01 ^b^
PHB Blend	1.43 ± 0.03 ^b^	−0.64 ± 0.03 ^e^	90.43 ± 0.04 ^a^	-	4.70 ± 0.02 ^e^	0.13 ± 0.03 ^b^
PHB Blend with CNCs	1.35 ± 0.02 ^b^	−0.28 ± 0.02 ^f^	90.07 ± 0.03 ^a^	0.52 ± 0.03 ^c^	3.43 ± 0.03 ^f^	0.13 ± 0.04 ^b^

a*: red/green coordinates (+a red, −a green); b*: yellow/blue coordinates (+b yellow, −b blue); L*: luminosity (+L luminous, −L dark); ΔE*: color differences; T: transparency; O: opacity. ^a–f^ Different letters in the same column indicate a significant difference among the samples (*p* < 0.05).

**Table 2:** Mechanical properties in terms of elastic modulus (E), tensile strength at yield (σ_y_), elongation at break (ε_b_), and toughness (T) of the different multilayers of poly(3-hydroxybutyrate-*co*-3-hydroxyvalerate) (PHBV) containing 8 mol.% (PHBV8) and 2 mol.% 3-hydroxyvalerate (PHBV2) and poly(3-hydroxybutyrate) with poly(butylene adipate-*co*-terephthalate) blend (PHB Blend) with and without cellulose nanocrystals (CNCs) in the transversal direction (TD) and machine direction (MD).

Sample	MD				TD			
	E (MPa)	σ_y_ (MPa)	ε_b_ (%)	T (mJ/m^3^)	E (MPa)	σ_y_ (MPa)	ε_b_ (%)	T (mJ/m^3^)
PHBV8	3223 ± 436 ^a^	24.5 ± 0.6 ^a^	2.6 ± 0.2 ^a^	0.45 ± 0.05 ^a^	2713 ± 469 ^a^	23.4 ± 1.6 ^a^	2.3 ± 0.3 ^a^	0.37 ± 0.08 ^a^
PHBV8 with CNCs	2304 ± 568 ^a,c^	22.4 ± 2.8 ^a,d^	2.1 ± 0.1 ^b^	0.33 ± 0.06 ^a^	2413 ± 364 ^a,d^	21.7 ± 1.3^a^	1.9 ± 0.2^a,c^	0.28 ± 0.06^a^
PHBV2	4267 ± 229 ^b^	39.0 ± 1.9 ^b^	1.4 ± 0.1 ^c^	0.33 ± 0.02 ^a^	4580 ± 317 ^b^	38.0 ± 0.5 ^b^	1.3 ± 0.1 ^b^	0.29 ± 0.03 ^a^
PHBV2 with CNCs	4789 ± 209 ^b^	44.9 ± 1.0 ^c^	0.19 ± 0.01 ^d^	0.59 ± 0.05 ^a^	4515 ± 132 ^b^	42.1 ± 2.2 ^b^	1.5 ± 0.1 ^b,c^	0.40 ± 0.01 ^a^
PHB Blend	1773 ± 138 ^c^	23.0 ± 0.1 ^d^	59.1 ± 39.2 ^e^	12.60 ± 3.10 ^b^	1624 ± 82 ^c^	20.8 ± 1.1 ^a^	61.0 ± 32.8 ^d^	12.40 ± 8.80 ^b^
PHB Blend with CNCs	2087 ± 332 ^c^	23.7 ± 2.7 ^a,d^	36.1 ± 14.7 ^e^	7.50 ± 1.60 ^b^	1937 ± 183 ^c,d^	20.9 ± 0.7 ^a^	10.6 ± 6.0 ^e^	1.84 ± 0.89 ^c^

^a–e^ Different letters in the same column indicate a significant difference among the samples (*p* < 0.05).

**Table 3:** Average thickness and permeance values in terms of water vapor permeance (WVP), d-limonene permeance (LP), and oxygen permeance (OP) of the multilayers of poly(3-hydroxybutyrate-*co*-3-hydroxyvalerate) (PHBV) containing 8 mol.% (PHBV8) and 2 mol.% 3-hydroxyvalerate (PHBV2) and poly(3-hydroxybutyrate) with poly(butylene adipate-*co*-terephthalate) blend (PHB Blend), with and without cellulose nanocrystals (CNCs).

Sample	Thickness (mm)	Permeance		
		WVP × 10^11^ (kg·m^−2^·Pa^−1^·s^−1^)	LP × 10^11^ (kg·m^−2^·Pa^−1^·s^−1^)	OP × 10^16^ (m^3^·m^−2^·Pa^−1^·s^−1^)
PHBV8	0.050 ± 0.002	11.47 ± 0.06 ^a^	13.91 ± 0.50 ^a^	57.81 ± 21.45 ^a^
PHBV8 with CNCs	0.055 ± 0.001	10.95 ± 0.05 ^a^	12.52 ± 0.33 ^b^	14.63 ± 3.34 ^b^
PHBV2	0.137 ± 0.006	0.90 ± 0.10 ^b^	2.02 ± 0.23 ^c^	6.37 ± 0.45 ^c^
PHBV2 with CNCs	0.140 ± 0.007	0.86 ± 0.02 ^b^	1.70 ± 0.22 ^c^	0.88 ± 0.07 ^d^
PHB Blend	0.150 ± 0.003	0.85 ± 0.03 ^b^	1.10 ± 0.20 ^d^	3.90 ± 0.91 ^e^
PHB Blend with CNCs	0.160 ± 0.004	0.82 ± 0.04 ^b^	0.79 ± 0.21 ^d^	1.12 ± 0.61 ^d^

^a–e^ Different letters in the same column indicate a significant difference among the samples (*p* < 0.05).
